# Integrated single-cell and bulk RNA-seq analysis reveals prognostic stemness genes in leiomyosarcoma

**DOI:** 10.3389/fonc.2025.1604413

**Published:** 2025-09-02

**Authors:** Aiju Lou, Yu Cai, Tuquan Zheng, Long Zhang, Yupeng Hu, Huachi Li, Lang Ying

**Affiliations:** ^1^ Department of Rheumatology, Liwan Central Hospital of Guangzhou, Guangzhou, China; ^2^ Guangzhou Key Laboratory of Spine Disease Prevention and Treatment, Department of Orthopaedic Surgery, The Third Affiliated Hospital of Guangzhou Medical University, Guangzhou, China; ^3^ Hubei Cancer Hospital, Tongji Medical College, Huazhong University of Science and Technology, Hubei Provincial Clinical Research Center for Colorectal Cancer, Wuhan Clinical Research Center for Colorectal Cancer, Wuhan, China; ^4^ The Sixth Affiliated Hospital of Nantong University, Yancheng Third People’s Hospital, The Yancheng School of Clinical Medicine of Nanjing Medical University, Yancheng, China

**Keywords:** leiomyosarcoma, prognostic markers, proliferation, stemness index, machine learning

## Abstract

**Introduction:**

Leiomyosarcoma (LMS) is a rare and aggressive soft tissue sarcoma with limited therapeutic options and poor prognosis. Identifying reliable prognostic markers and therapeutic targets is critical for improving personalized treatment strategies.

**Methods:**

We integrated single-cell RNA sequencing (scRNA-seq) data of LMS from the Gene Expression Omnibus (GEO) with bulk RNA-seq and clinical data from The Cancer Genome Atlas (TCGA). Malignant cells were identified using machine learning approaches, and their stemness index was calculated. Cells were stratified into high and low stemness index groups, and differential gene expression analysis was performed. Prognostic markers were identified through a sequential pipeline: univariate Cox regression to screen candidate genes, Lasso regression for feature selection, and multivariate Cox regression for model construction and survival analysis.

**Results:**

Cells with a high stemness index exhibited a more complex tumor immune microenvironment (TIME) and enhanced intercellular interactions compared to those with a low stemness index. Differential expression analysis identified genes distinguishing high versus low stemness cells. Through the regression pipeline, six prognostic markers were identified: BOP1, CTBP1, DSE, PMSD10, SRPK1, and HACD4. These markers were significantly associated with tumor cell proliferation and patient survival outcomes.

**Discussion:**

Our findings suggest that stemness-related heterogeneity in LMS shapes the tumor immune microenvironment and contributes to disease progression. The six identified prognostic markers not only provide insights into the molecular mechanisms of LMS but also represent potential therapeutic targets for personalized treatment.

## 1 Introduction

Leiomyosarcoma (LMS) is a rare and highly aggressive subtype of soft tissue sarcoma (STS), characterized by smooth muscle differentiation and a high metastatic potential. LMS can occur in various anatomical locations, including the uterus, gastrointestinal tract, extremities and retroperitoneum, with clinical behaviors that differ based on the primary site of origin. LMS can occur in various anatomical locations, including the uterus, gastrointestinal tract, extremities, and retroperitoneum, with clinical behaviors that differ based on the primary site of origin. Despite advancements in multimodal treatment strategies, the prognosis for patients with advanced LMS remains poor due to high recurrence rates and resistance to conventional chemotherapy (1–7).

Cancer remains a leading public health challenge in the United States, with an estimated 2,001,140 new cancer cases and 611,720 cancer-related deaths expected in 2024, making it the second leading cause of death after heart disease (8). Among the many cancer subtypes, leiomyosarcoma (LMS) represents a rare but highly aggressive soft tissue sarcoma subtype with a notably poor prognosis, particularly in its advanced stages (9). According to current clinical reviews, LMS often demonstrates high recurrence rates and substantial resistance to conventional chemotherapy and radiotherapy, which limits the efficacy of standard treatment strategies (10).

Recent therapeutic approaches have begun to incorporate personalized medicine and targeted therapy, but their effectiveness in LMS remains limited. Despite innovations such as immune checkpoint blockade and cell-based therapies, substantial heterogeneity and immune evasion mechanisms in tumors like LMS continue to present therapeutic challenges. These limitations underscore the need to better understand tumor biology and identify novel biomarkers to improve risk stratification and guide treatment.Therefore, identifying reliable prognostic markers is critical for improving patient stratification and guiding personalized treatment approaches (11).

However, clinical cohorts and multi-omics data specific to LMS remain relatively scarce, limiting our understanding of its pathogenesis and hindering the optimization of treatment modalities, particularly in the field of precision medicine. With the rapid development of technologies such as single-cell RNA sequencing (scRNA-seq), single-cell multi-omics, machine learning, and deep learning, there has been significant progress in the study of tumor heterogeneity (12, 13), the complex cellular interactions within the tumor immune microenvironment (TIME), and the identification of novel therapeutic targets. These technological advancements have provided deeper insights into the mechanisms and treatment strategies or LMS (14–16). Notably, research on ULMS, aided by genomics and molecular markers, has opened new avenues for its diagnosis and treatment (17, 18).

Recent studies have highlighted the significant role of tumor proliferation and the TIME in LMS progression and patient prognosis. A high mitotic index and uncontrolled cellular proliferation are closely associated with poor prognosis, underscoring the importance of molecular markers that predict tumor aggressiveness (15, 17). Single-cell RNA sequencing, as a powerful tool, allows for unprecedented precision in deciphering tumor heterogeneity, enabling the identification of malignant cell populations with distinct transcriptional programs. By integrating scRNA-seq data from Gene Expression Omnibus (GEO) and bulk RNA-seq and clinical data from The Cancer Genome Atlas (TCGA), new prognostic markers can be identified to optimize risk assessment and guide treatment decisions (19–21).

In recent years, integrative transcriptomic approaches combining single-cell and bulk RNA-sequencing data have enabled deeper exploration of tumor heterogeneity and prognostic marker discovery. The stemness index (mRNAsi), derived using a one-class logistic regression (OCLR) model, quantifies the similarity between tumor cells and stem cells, serving as an indicator of cellular plasticity and potential tumor aggressiveness (22). This metric has proven valuable in stratifying tumors based on their dedifferentiation status and therapeutic resistance potential.

To evaluate associations between gene expression and patient survival, Cox proportional hazards regression provides a widely used statistical framework for time-to-event analysis. When combined with Lasso (Least Absolute Shrinkage and Selection Operator) regression, a regularization method that enhances model interpretability by selecting the most informative variables, this approach allows for the construction of robust prognostic models (23, 24). Applying these methods in the context of LMS may offer new insights into key molecular drivers of tumor progression and support the development of personalized therapeutic strategies.

## 2 Methods

### 2.1 Data collection and processing

For the TCGA-SARC cohort, we retrieved normalized RNA sequencing data (expressed as Fragments Per Kilobase per Million, TPM) for 263 sarcoma (SARC) patients and 2 normal samples, along with corresponding clinical data from TCGA database (https://portal.gdc.cancer.gov/). Additionally, single-cell RNA sequencing (scRNA-seq) (25) data for leiomyosarcoma were retrieved from the GSE212527 (26) dataset in the GEO database (https://www.ncbi.nlm.nih.gov/). This dataset includes three sarcoma and four leiomyosarcoma samples, from which we selected four leiomyosarcoma samples for further analysis.

### 2.2 scRNA-seq data processing and cell type annotation

The “Seurat” R package (v4.4.0) (27) was used to analysis single-cell data. Single-cell data were read with a minimum feature count of 200 (min.features = 200) and a minimum cell count of 3 (min.cells = 3). Cells with high mitochondrial gene expression (>20%) were excluded to filter out low-quality cells. Mitochondrial genes (those starting with “MT”) and ribosomal protein genes (“RPS” and “RPL”) were also removed. The single-cell data were then integrated using the Harmony (28) algorithm to remove batch effects. Finally, PCA was applied for dimensionality reduction using the top 50 principal components, followed by UMAP for further unsupervised clustering of the cells.

Each cell cluster was annotated using characteristic genes corresponding to specific cell types, as obtained from the CellMarker 2.0 database (29) (http://bio-bigdata.hrbmu.edu.cn/CellMarker). Marker genes for each cluster were identified using the FindAllMarkers function (test.use = “wilcox”, min.pct = 0.1) in the Seurat package with default parameters, and those with |log2FC| > 0.25 and adjusted p-value< 0.05 were considered significant. Cell type identities were assigned by cross-referencing these cluster-specific marker genes with curated cell type markers in the CellMarker database. To distinguish immune cell subtypes, well-established canonical markers were used—for example, CD2, CD3E and CD8A for T cells, KLRC1, KLRD1 and GNLY for NK cells.

### 2.3 Aneuploid cells identification based on machine leaning

To distinguish malignant tumor cells from non-malignant cells in the tumor microenvironment, we applied CopyKAT (Copy Number Karyotyping of Tumors) (30), a computational tool that infers genome-wide DNA copy number variations (CNVs) from single-cell RNA-seq data. CopyKAT integrates a Bayesian segmentation algorithm to detect large-scale chromosomal gains and losses at approximately 5 megabase (Mb) resolution. CopyKAT uses unsupervised clustering based on genome-wide CNV patterns to classify cells as diploid or aneuploid, reflecting non-malignant and malignant states, respectively. Aneuploidy, a hallmark of over 90% of human cancers, serves as the key distinguishing feature. Cells with extensive CNV profiles indicative of aneuploidy are classified as malignant, while those exhibiting diploid-like CNV patterns are considered non-malignant stromal or immune cells. This analysis enables robust identification of malignant subpopulations within complex tumor tissues.

### 2.4 Classification of aneuploid cells into high and low stemness groups

We identified aneuploid cell clusters and performed reclustering. Then, we used machine learning to calculate the stemness index for each cell. We trained and built a model using the one class linear regression (OCLR) (22) on human stem cell data from progenitor cell biology consortium (PCBC, https://www.synapse.org). The model was implemented using the gelnet R package (31), with elastic net regularization applied (α = 0.5) to balance L1 and L2 penalties. The regularization parameter (λ) was optimized via 5-fold cross-validation. Only pluripotent stem cells were used as the positive class to define the stemness signature. The stemness signature was derived from 11,774 samples, and 500 genes with the highest coefficients from the OCLR model were retained to calculate the mRNAsi. After training, the model generated a stemness score vector for all genes, and the stemness index (mRNAsi) for each single cell was calculated as the Spearman correlation between this gene score vector and the gene expression profile of the cell. The stemness score was visualized using a UMAP (32) plot, which enabled the classification of cells into high and low stemness groups.

### 2.5 Differential cell communication analysis

For aneuploid cells in the high and low stemness groups, CellChat (33) algorithm was used to calculate their cell-cell communication with other cells. Communication strength between cell groups was quantified based on communication probability scores derived from known ligand–receptor interactions. The computeCommunProb function was applied to calculate interaction probability for each pair of cell types, and significant interactions were retained with default statistical thresholds (p< 0.05). Interaction strength was further aggregated using aggregateNet, generating total interaction number and weight matrices. Differential communication strength between High mRNAsi and Low mRNAsi groups was assessed using netVisual_diffInteraction and rankNet, and pathway activity was validated via dot plots of ligand–receptor gene expression using plotGeneExpression function. This analysis helped to explore the differences in the immune microenvironment between these two cell types.

### 2.6 Differential gene identification and pathway analysis

Differential gene identification between high and low stemness groups was performed using the FindMarkers function (test.use = “wilcox”, min.pct = 0.1) in Seurat V4.4.0 (27) with default parameters, and those with |log2FC| > 0.5 and adjusted p-value< 0.05 were considered significant. Additionally, gene set variation analysis (GSVA) (34) was applied for differential pathway analysis.

### 2.7 Construction of prognostic model and survival analysis

Differential genes between the high and low stemness groups were initially screened using univariate Cox regression. Then, Lasso regression (35) was applied using the glmnet package in R with alpha = 1, corresponding to a pure L1 penalty. This approach promotes model sparsity and facilitates the selection of the most prognostically relevant genes. The optimal regularization parameter (lambda.min) was determined via 10-fold cross-validation and was found to be 0.056. Finally, multivariate Cox regression (36) was used to identify a gene set that significantly contributes to prognosis. The stemness risk score was calculated using the following formula:


$$RiskScore=\;\sum_{i=1}^{n}\left(Cox\;coefficient_{i}\times Gene\;Expression_{i}\right)$$

To assess the predictive performance of the prognostic model, we constructed time-dependent Receiver Operating Characteristic (ROC) (37–40) curves and calculated the area under the curve (AUC) at 1-, 3-, and 5-year intervals. AUC values were used to quantify the model’s discriminative ability in predicting overall survival, with higher AUC indicating better predictive performance.

### 2.8 Immune infiltration analysis and interaction network prediction of prognostic genes

For the final selected prognostic genes, TIMER2.0 (41) was used to analyze their tumor immune infiltration. We performed immune cell deconvolution based on bulk RNA-seq data from the TCGA-SARC cohort. Specifically, the CIBERSORT algorithm was applied using the LM22 signature matrix, which defines the expression profiles of 22 distinct human hematopoietic cell phenotypes, including B cells, T cells, NK cells, macrophages, and dendritic cells. Additionally, GeneMANIA (42) was applied to predict the interaction network and regulatory genes associated with these risk genes.

### 2.9 Cell culture and gene silencing

The human leiomyosarcoma cell line SK-LMS-1 was cultured in RPMI-1640 medium (Gibco) supplemented with 10% fetal bovine serum (FBS) (Gibco) and 1% penicillin-streptomycin (Gibco) at 37 °C in a humidified atmosphere containing 5% CO2. Cells were transfected with siRNA targeting BOP1, CTBP1, DSE, PMSD10, and SRPK1 using Lipofectamine RNAiMAX transfection reagent (43) (Thermo Fisher) according to the manufacturer’s instructions. The control group was transfected with a non-targeting siRNA (siCon). The transfection was carried out with a final concentration of 1.5 µL Lipofectamine RNAiMAX per 5 pM siRNA, and the incubation time was 24 hours. The siRNA sequences targeting BOP1, CTBP1, DSE, PMSD10, and SRPK1 are provided in **Supplementary Table S1**. Gene silencing efficiency was verified by qRT-PCR. The primer sequences used for qRT-PCR amplification are provided in **Supplementary Table S2**.

### 2.10 Scratch assay

Cell migration was assessed using the scratch assay. After transfection, cells were cultured to confluence in 6-well plates, and a wound was created using a sterile pipette tip. The cells were then washed with PBS and cultured in serum-free medium for 24 hours. Images of the scratch were captured at 0 and 24 hours using a light microscope (44) (Leica). Migration distance was quantified by measuring the width of the wound at different time points.

### 2.11 Transwell migration assay

Cell migration was assessed using Transwell inserts (Corning, 8 µm pore size). After transfection, 1 × 10^5^ cells were seeded into the upper chamber in serum-free medium, and the lower chamber was filled with medium containing 10% FBS as a chemoattractant. After 24 hours, cells that migrated through the membrane were fixed with methanol and stained with crystal violet. Migrated cells were counted in five random fields per membrane under a light microscope.

### 2.12 Colony formation assay

For colony formation assays, 500 cells per well were seeded in 6-well plates after transfection. After 10–14 days, cells were fixed with 4% paraformaldehyde and stained with crystal violet. Colonies consisting of at least 50 cells were counted under a light microscope.

### 2.13 Quantification of assays and statistical analysis

Quantification of migration and colony formation was performed using ImageJ (45) software. For the scratch assay, the percentage of wound closure was calculated based on the initial and final widths of the wound. For the Transwell migration and colony formation assays, the number of migrated cells or colonies was counted in five random fields per well.

Data were analyzed using GraphPad Prism 8 (GraphPad Software). Statistical significance was determined using one-way ANOVA followed by Tukey’s *post hoc* test for multiple comparisons. A p-value of< 0.05 was considered statistically significant.

## 3 Results

### 3.1 Single-cell RNA sequencing data analysis of LMS samples

**Figure 1:**
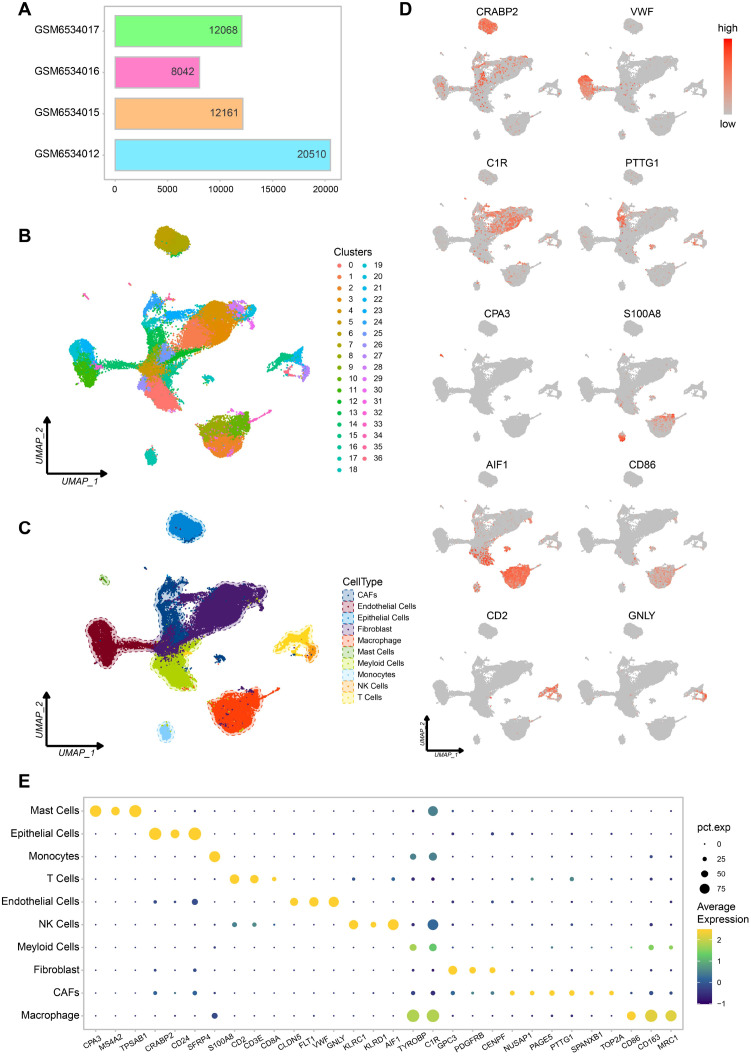
Different cell types in scRNA-seq data of leiomyosarcoma. **(A)** Cell count statistics for each sample. **(B)** Cell clustering UMAP plot. **(C)** Cell types UMAP plot. **(D)** UMAP plot of marker genes for each cell type, Each plot displays one marker gene, with expression intensity represented by a gray-to-red color scale (low to high). **(E)** Bubble plot of marker gene expression for each cell type.

We integrated single cell RNA sequencing data from four LMS samples (GSM6534012, GSM6534015, GSM6534016, and GSM6534017) and remove mitochondrial genes, ribosomal genes, and batch effects between samples. The final integrated dataset consisted of 52,781 cells (**Figure 1A**). We then performed unsupervised clustering on this dataset, resulting in 37 distinct cell clusters (**Figure 1B**). Cell clusters were annotated using marker genes corresponding to human cell types from the CellMarker 2.0 database, leading to the identification of 10 major cell types (**Figure 1C**). As shown in the figure, epithelial cells and fibroblasts make up the majority. Each cell type was supported by 2–3 marker genes, and the correct and reasonable annotation of these cell types was further validated by gene expression UMAP plots (**Figure 1D**).

Among these cells, we also identified cancer associated fibroblasts (CAFs) and immune cells such as macrophages. This suggests that the dataset provides a rich variety of cell types and a stable immune microenvironment, which will be valuable for our subsequent research (**Figure 1E**).

### 3.2 Aneuploid cells with high stemness demonstrate increased invasiveness

**Figure 2:**
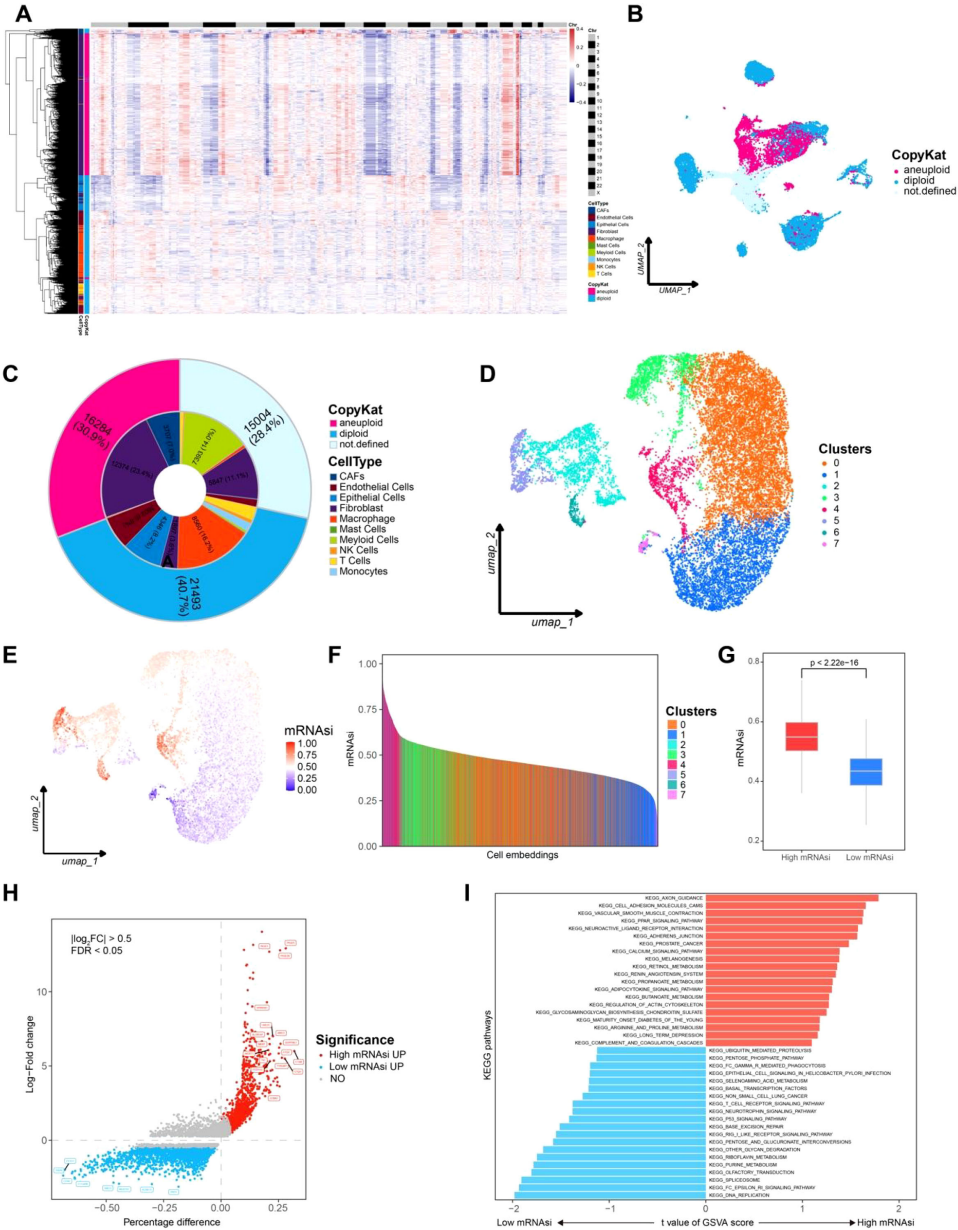
Aneuploid cells identification and mRNAsi calculation for each cells. **(A)** CNVs heatmap of CopyKat, the top black-and-white bar indicates chromosome boundaries from Chr 1 to Chr X. Annotations on the left represent predicted cell types, while the right bar indicates CopyKat-classified ploidy states (pink for aneuploid, cyan for diploid). **(B)** Aneuploid cell clusters UMAP plot. **(C)** Pie chart representing the composition of aneuploid cells. **(D)** UMAP plot for cell clustering of aneuploid cells. **(E)** UMAP plot showing the distribution of mRNAsi across cells. **(F)** mRNAsi rank plot. **(G)** Box plot comparing high and low mRNAsi groups. **(H)** Differential expressed genes between high and low mRNAsi groups, with red indicating high mRNAsi and blue indicating low mRNAsi. **(I)** GSVA analysis comparing high and low mRNAsi groups, with red indicating high mRNAsi and blue indicating low mRNAsi.

We applied CopyKat algorithm to all cells to calculate genome wide copy number variations (CNVs) based on single cell transcriptomic data. Since aneuploid CNVs are the most common genetic alterations in human tumors (occurring in approximately 88% of cases), while stromal cell types with diploid genomes typically lack such alterations, CNVs analysis allows for the identification of aneuploid cells, which correspond to tumor cells (**Figure 2A**). Through CNVs-based classification, we identified 16,284 tumor cells (30.9%). Notably, the identified tumor cells were predominantly CAFs and fibroblasts, whereas diploid cells included endothelial cells, immune cells, and other normal cell types (**Figures 2B, C**). This classification highlights the distinct genomic alterations present in malignant versus normal stromal cells within the tumor environment.

The identified tumor cells were further selected for reclustering, resulting in seven distinct cell clusters. A model based on stem cell gene expression data was trained to calculate the mRNA stemness index (mRNAsi) for each tumor cell. The mRNAsi scores were then mapped onto a UMAP plot for visualization (**Figure 2D**). Notably, clusters 2, 3, 4, 5, and 6 exhibited high mRNAsi levels. Based on the distribution of mRNAsi scores, tumor cells were classified into high mRNAsi and low mRNAsi groups for subsequent analyses (**Figures 2E–G**).

For the high and low mRNAsi groups, differential gene expression analysis was performed using the FindMarkers function in Seurat package (p-value< 0.05, log_2_(FC) = 0.25). Genes specifically upregulated in the high mRNAsi group included MKI67, MGST1, and ALOX5AP, which are associated with lipid metabolism, endoplasmic reticulum stress, and tumor progression. In contrast, genes highly expressed in the low mRNAsi group, such as TRDN and FXYD1, were involved in sodium-calcium channel homeostasis, suggesting a distinct cellular regulatory mechanism (**Figure 2H**). These findings indicate that high stemness tumor cells may possess greater invasive potential.

GSVA analysis revealed significant enrichment of the KEGG_AXON_GUIDANCE pathway in the high mRNAsi group. This pathway integrates Rho GTPase, PI3K/AKT, and MAPK/ERK signaling, which collectively promote tumor invasion and migration. Meanwhile the KEGG_DNA_REPLICATION pathway was significantly enriched in low mRNAsi group, a pathway that is crucial for maintaining genomic integrity and is highly conserved across organisms(**Figure 2I**). These results suggest that high stemness tumor cells in LMS exhibit stronger invasive capabilities, further supporting the hypothesis that stemness related gene expression contributes to tumor aggressiveness.

### 3.3 Aneuploid cells with high stemness demonstrate a more complex environment and invasion potential

**Figure 3:**
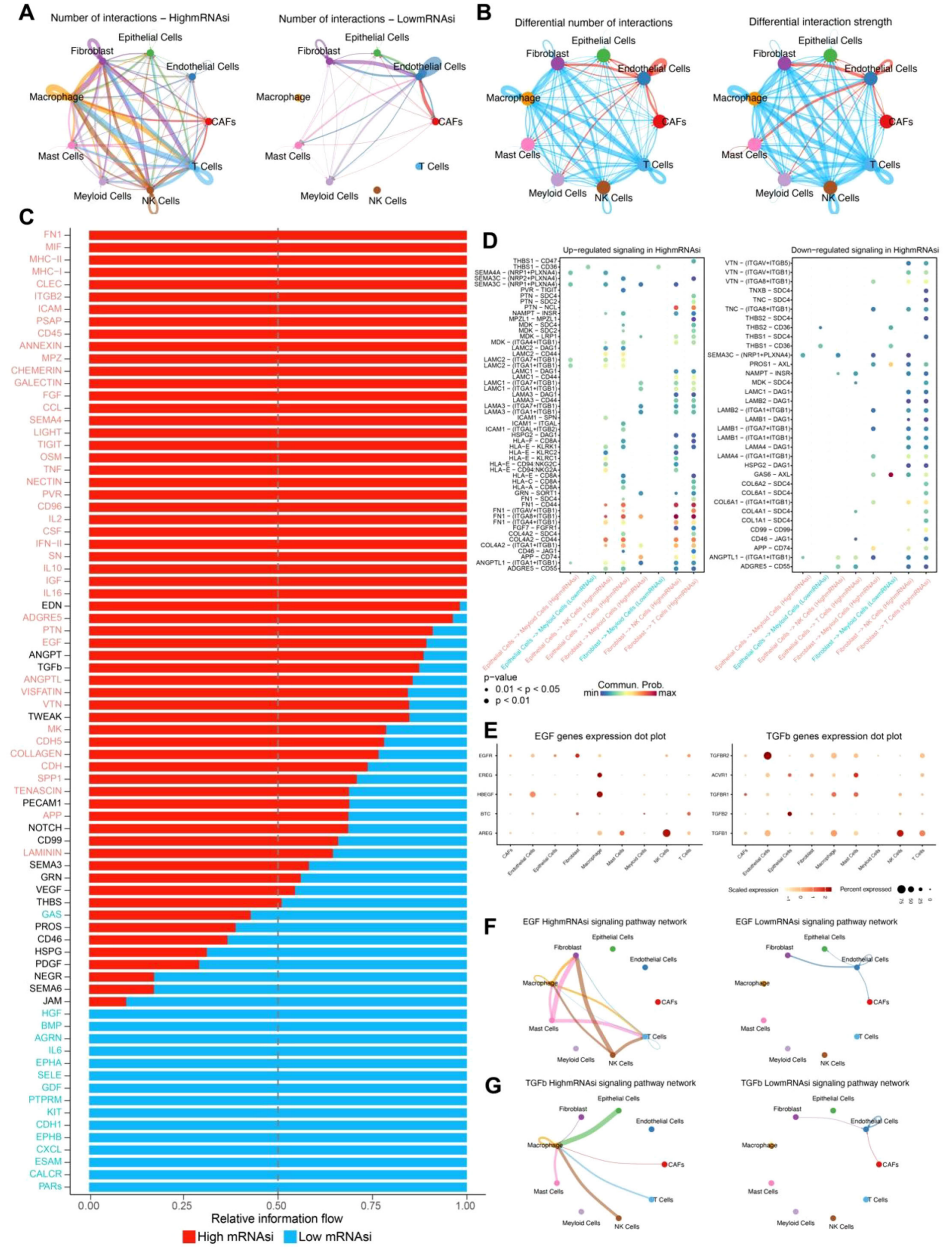
Cell communication between high and low mRNAsi groups. **(A)** Number of interactions between high and low mRNAsi groups. **(B)** Differential number and strength of interactions between high and low mRNAsi groups. **(C)** Relative signal information flow between high and low mRNAsi groups, with red indicating high mRNAsi and blue indicating low mRNAsi. **(D)** Bubble plot of differential signaling between high and low mRNAsi groups. **(E)** Heatmap of gene expression levels for the EGF and TGF-β signaling pathways across different cell types. **(F)** EGF signaling pathway network between high and low mRNAsi groups. **(G)** TGF*β* signaling pathway network between high and low mRNAsi groups.

Analysis of cell-cell communication between the high and low mRNAsi groups revealed a significantly higher number of interactions among epithelial cells, fibroblasts, and immune cells such as macrophages in the high mRNAsi group compared to the low mRNAsi group (**Figure 3A**). Moreover, fibroblast-immune cell interactions were markedly stronger in the high mRNAsi group, whereas epithelial-immune cell interactions were more prominent in the low mRNAsi group (**Figure 3B**). These findings suggest that the tumor immune microenvironment in the high mRNAsi group is more complex and may have an increased risk of immune evasion.

Further comparison of differential signaling flows between the two groups revealed a significant upregulation of signaling pathways associated with tumor metastasis, immune evasion, and inflammatory response, including TGF*β*, EGF, and IL2, in the high mRNAsi group (**Figure 3C**). Additionally, ligand-receptor interactions were more abundant in this group, indicating a higher level of cellular communication complexity. Notably, several ligand-receptor pairs associated with tumor progression were identified between fibroblasts and immune cells, including FN1-(ITGA8+ITGB1), FN1-CD44, COL4A2-CD44, and ANGPTL1-(ITGA8+ITGB1) (**Figure 3D**). These ligand-receptor interactions play a crucial role in tumor angiogenesis, cell migration, invasion, and immune evasion, further supporting the hypothesis that high mRNAsi tumor cells possess greater invasive and metastasis potential.

To further validate the inferred cell–cell communication patterns, we analyzed the expression of key ligand–receptor pairs involved in the EGF and TGFβ signaling pathways across different cell types. Several EGF pathway genes, including AREG, HBEGF, and EGFR, exhibited cell type-specific expression patterns. Notably, AREG and HBEGF were highly expressed in NK cells and macrophages, respectively, whereas EGFR showed broader but moderate expression across multiple stromal and immune cell populations. These findings suggest that NK cells and macrophages may act as major sources of EGF ligands in the tumor microenvironment. For the TGFβ signaling pathway, high expression of TGFBR2, TGFBR1, and TGFβ1 was observed in CAFs, macrophages, and myeloid cells, indicating that stromal and myeloid populations are both senders and receivers of TGFβ signals. Particularly, TGFBR2 was abundantly expressed in CAFs, highlighting their potential role in receiving and propagating TGFβ signaling (**Figure 3E**). Together, these ligand–receptor expression patterns confirm the cell type-specific activation of EGF and TGFβ signaling pathways and support the functional relevance of these interactions predicted by CellChat.

Analysis of TGF*β* and EGF signaling networks demonstrated that both the interaction frequency and intensity of TGF*β* and EGF signaling factors were significantly higher in the high mRNAsi group than in the low mRNAsi group (**Figures 3F, G**). These findings provide strong evidence for the role of these signaling pathways in the development and progression of leiomyosarcoma, offering valuable insights for potential targeted therapeutic strategies.

### 3.4 High risk stemness genes are linked to poor prognosis

**Figure 4:**
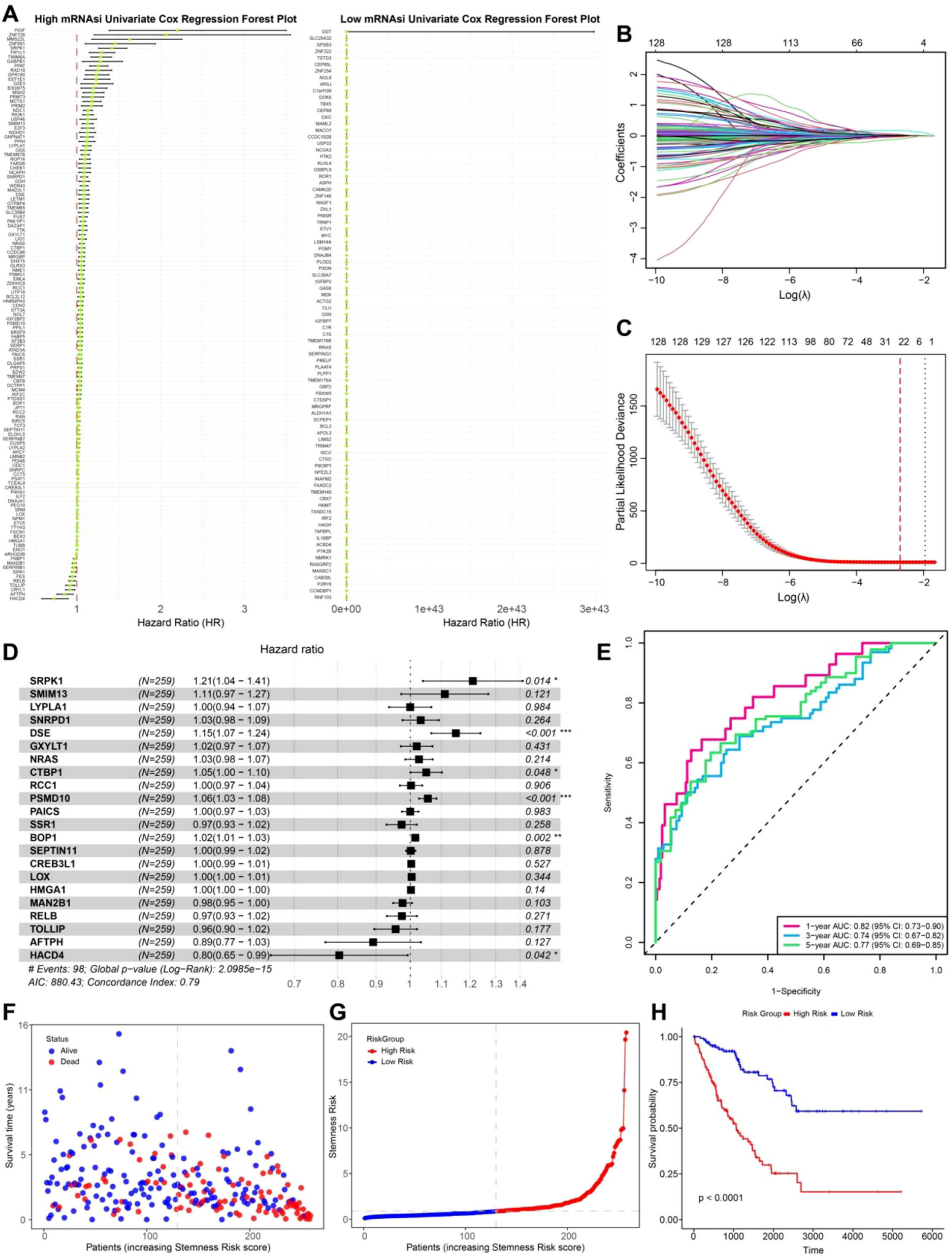
Prognostic genes selection and survival analysis. **(A)** Univariate Cox regression forest plot of high and low mRNAsi groups. **(B)** Lasso regression coefficient pathway plot. Each curve represents the trajectory of a coefficient for each predictor variable. The vertical axis shows the value of the coefficient, while the lower horizontal axis represents log(λ). **(C)** Lasso regression cross-validation curve. The vertical axis represents the binomial deviance, with the dashed line on the left indicating the lowest error, and the dashed line on the right representing the model with fewer features. **(D)** Multivariate Cox regression forest plot. **(E)** Time-dependent ROC curve (1, 3, 5 years). **(F)** Survival status scatter plot. **(G)** Risk score ranking plot. **(H)** Survival curve plot.

Survival analysis was conducted differentially expressed genes (DEGs) between the high and low mRNAsi groups in patients from the TCGA-SARC cohort (n = 263). Univariate Cox regression analysis of these DEGs revealed that genes specifically upregulated in the high mRNAsi group were predominantly risk associated genes, whereas those upregulated in the low mRNAsi group were not significantly associated with poor prognosis (**Figure 4A**). To further refine prognostic gene selection, Lasso regression (**Figures 4B, C**) was applied, at lambda value 0.056, a total of 24 feature genes with non-zero coefficients were selected for subsequent multivariate Cox regression analysis, ultimately identifying six prognostically relevant genes: SRPK1, DSE, CTBP1, PSMD10, BOP1, and HACD4. Among them, SRPK1, DSE, CTBP1, PSMD10, and BOP1 were classified as risk genes negatively affecting prognosis, whereas HACD4 was identified as a protective gene associated with better survival outcomes (**Figure 4D**).

A stemness risk score was then constructed based on the Cox regression coefficients of these six genes. The predictive performance of this model was validated using time-dependent ROC curves at 1, 3, and 5 years, yielding AUC values of 82%, 77%, and 79%, respectively, indicating a strong prognostic capability (**Figure 4E**). Patients were further stratified into high risk and low risk groups based on the median stemness risk score. The analysis revealed that patients with higher risk scores exhibited a significantly higher mortality rate, and a higher stemness risk score correlated with a worse prognosis (**Figures 4F, G**). Kaplan-Meier (KM) survival analysis further confirmed that the high risk group had significantly poorer survival outcomes compared to the low risk group (**Figure 4H**). This study systematically filtered differential genes between high and low mRNAsi groups, leading to the development of a prognostic model that successfully identified genes associated with tumor cell migration and metastasis in LMS. These findings provide new insights into the poor prognosis of LMS and highlight potential therapeutic targets for precision medicine in clinical applications.

### 3.5 Risk genes exhibit a complex immune infiltration and regulatory network

**Figure 5:**
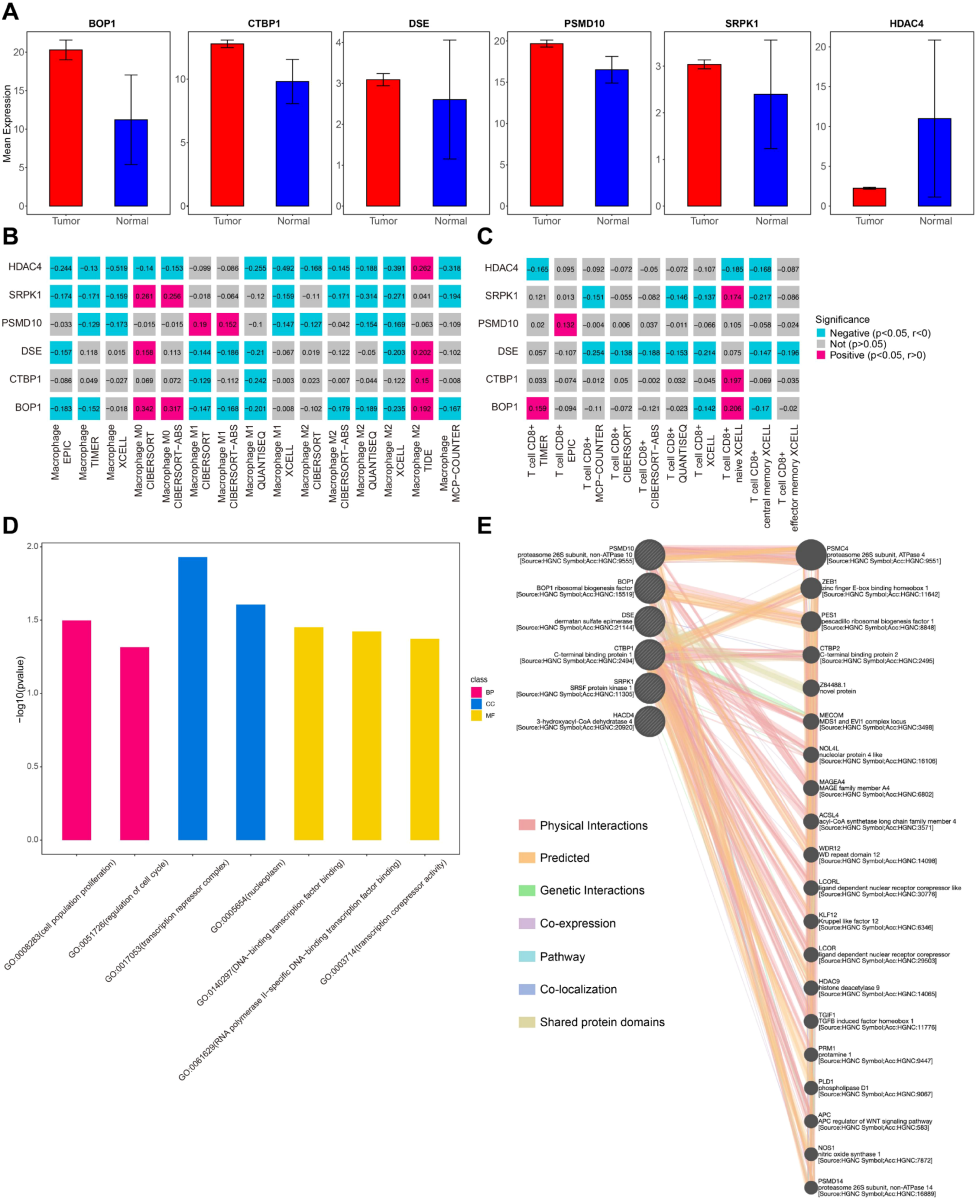
Risk genes immune infiltration and regulatory analysis. **(A)** Bar plot of the mean expression of 6 risk genes. **(B)** Macrophage infiltration analysis of 6 risk genes. **(C)** T cells infiltration analysis of 6 risk genes. **(D)** GO analysis of 6 risk genes. **(E)** Regulatory network of 6 risk genes.

To investigate the expression patterns of the six prognostic genes, a bar plot was used to compare their expression levels between tumor patients and normal samples in the TCGA-SARC cohort. The analysis revealed that risk genes (BOP1, CTBP1, DSE, SRPK1, and PMSD10) were significantly upregulated in tumor samples, whereas the protective gene HACD4 was highly expressed in normal samples (**Figure 5A**).

To further explore the immune infiltration landscape, the TIMER2.0 tool was used to assess the association of these prognostic genes with immune cell populations. The results showed that these genes were positively correlated with M0 macrophages and naive CD8+ T cells, while exhibiting a negative correlation with other macrophage subtypes (**Figures 5B, C**). These findings suggest that further classification of macrophages within the LMS tumor microenvironment could provide deeper insights into their functional roles in tumor progression. Functional enrichment analysis indicated that these genes were significantly involved in pathways related to cell invasion and the cell cycle, both of which are critical for tumor progression and metastasis (**Figure 5D**).

Additionally, GeneMANIA was used to predict the gene regulatory network, revealing a complex interaction network involving several key cancer-associated genes, such as TGIF1, PLD1, and PMSC4 (**Figure 5E**). These genes, through their interactions with the identified prognostic genes, may play a pivotal role in promoting tumor cell invasion and metastasis, further highlighting their potential as therapeutic targets in LMS.

### 3.6 Stemness prognostic genes drive LMS cell migration and invasion

**Figure 6:**
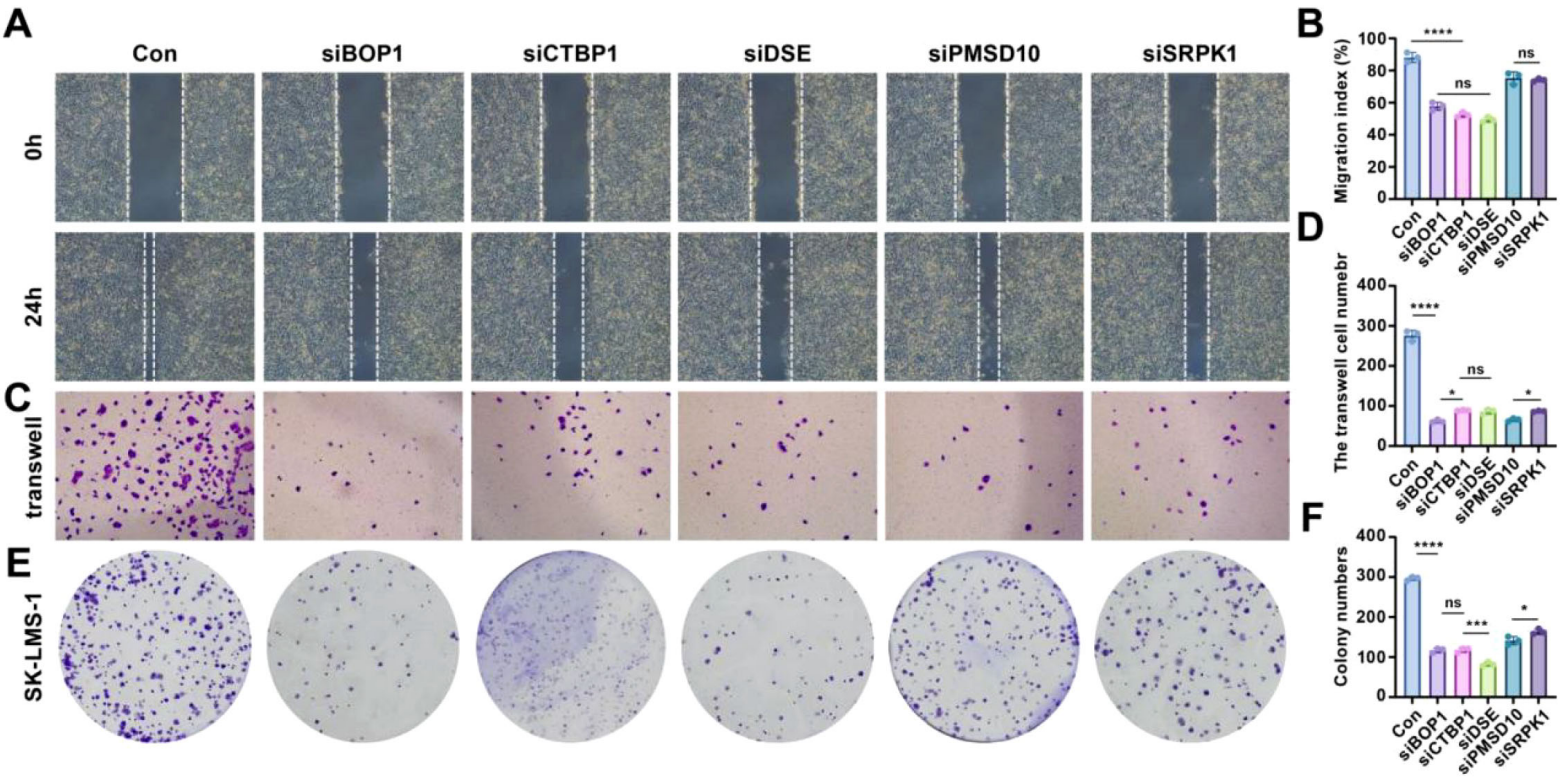
Effects of knockdown of stemness-related genes on cell behavior in LMS. **(A)** Scratch assay showing cell migration in control and gene knockdown conditions. **(B)** Relative quantitative analysis of cell migration. **(C)** Transwell migration assay for control and gene knockdown groups. **(D)** Quantitative analysis of Transwell migration results. **(E)** Colony formation assay comparing control and gene knockdown conditions. **(F)** Quantification of colony numbers in each group. Statistical significance is indicated by asterisks: *p< 0.05, ***p< 0.001, ****p< 0.0001, ns: no significant difference.

To assess the role of stemness-related genes in the migration, invasion, and colony formation of LMS cells, several functional assays were conducted. Firstly, the knockdown efficiencies for BOP1, CTBP1, DSE, PMSD10, and SRPK1 were 83.09%, 78.72%, 78.36%, 72.43%, and 79.59%, respectively (see **Supplementary Figures S1-S5** for details). Scratch assays revealed that silencing BOP1, CTBP1, DSE, PMSD10, and SRPK1 significantly reduced cell migration compared to control cells (**Figure 6A**). Quantitative analysis of migration showed a marked decrease in the migration index for all gene knockdown conditions, with significant differences observed (p< 0.05, *p< 0.001; **Figure 6B**). The Transwell migration assay confirmed these findings, with fewer cells migrating through the membrane in the knockdown groups compared to the control (**Figure 6C**). Quantification of migrated cells further demonstrated a significant reduction in cell migration upon gene knockdown, supporting the hypothesis that these genes influence cell invasiveness (*p< 0.001; **Figure 6D**). Colony formation assays also showed that silencing BOP1, CTBP1, DSE, PMSD10, and SRPK1 resulted in a significant reduction in colony numbers compared to the control group (**Figure 6E**). Quantification of colony formation further confirmed that the number of colonies formed was significantly lower in the knockdown groups (p< 0.05, *p< 0.001; **Figure 6F**).

These results suggest that the stemness-related genes BOP1, CTBP1, DSE, PMSD10, and SRPK1 play critical roles in promoting cell migration, invasion, and colony formation, highlighting their potential as targets for therapeutic intervention in LMS.

## 4 Discussion

### 4.1 Prognostic implications of stemness in LMS

In this study, we utilized scRNA-seq data from LMS samples and bulk RNA-seq data from the TCGA-SARC cohort to identify key prognostic markers related to tumor proliferation and stemness. Because TCGA bulk RNA-seq datasets have been widely used in cancer research, they are inherently limited by technical and biological biases. As highlighted in recent reviews (46, 47), bulk data represents an average of gene expression across heterogeneous cell populations, masking critical cell type specific signals. This can lead to inaccurate identification of prognostic markers, particularly in tumors with complex microenvironments like LMS. Additionally, batch effects, platform differences, and variability in tumor purity further complicate interpretation. To address these challenges, we integrated single-cell RNA-seq data, which enables resolution of gene expression at the individual cell level. This approach allows for more accurate identification of malignant versus stromal or immune cells, characterization of tumor cell heterogeneity, and precise assessment of the tumor immune microenvironment. In the context of LMS, where stromal infiltration and immune composition vary widely, single-cell analysis provides a more reliable framework for identifying clinically relevant biomarkers and understanding tumor biology.

Using machine learning algorithms, we classified cells based on their stemness index and explored differences in TIME complexity between high and low stemness groups. The findings suggest that high mRNAsi tumor cells exhibit a more complex immune microenvironment, potentially contributing to greater invasiveness and tumor progression. Our findings that high mRNAsi tumor cells are associated with a more complex immune microenvironment in LMS are consistent with previous studies in other cancer types. For example, similar associations were observed in gastric cancer, lung adenocarcinoma, esophageal cancer, and gastrointestinal stromal tumors, where stemness features were linked to immune infiltration and tumor aggressiveness (10, 48–50). By integrating both single-cell and bulk RNA-seq data, we identified six key prognostic genes (BOP1, CTBP1, DSE, PMSD10, SRPK1, and HACD4), which were associated with poor prognosis and tumor cell proliferation. Our analysis revealed that the high mRNAsi group was characterized by stronger cell-cell interactions and increased expression of risk genes. These high-stemness cells showed a more active signaling landscape, with pathways related to tumor invasion, metastasis, and immune evasion such as TGFβ and EGF signaling pathways. In contrast, the low mRNAsi group exhibited a distinct cellular regulation mechanism with a lower degree of immune and stromal cell interactions. This dichotomy suggests that high-stemness tumor cells might not only be more aggressive but also present a resistant phenotype to conventional therapies.

The identification of prognostic markers through Cox regression and Lasso regression further highlighted the significant role of the risk genes BOP1, CTBP1, DSE, SRPK1, and PMSD10, which were upregulated in high mRNAsi tumor cells. In contrast, HACD4 emerged as a protective gene, associated with a better prognosis and lower risk. The prognostic value of these genes was validated through survival analysis, with a stemness risk score serving as a robust predictive tool for patient stratification. Additionally, our study explored the immune infiltration and cell communication networks in LMS. Using TIMER2.0 and GeneMANIA, we identified a complex regulatory network involving key cancer-related genes such as TGIF1, PLD1, and PMSC4, which influence the tumor’s ability to evade immune responses and metastasize. The results suggest that these genes contribute significantly to tumor progression through their interaction with immune cells, specifically macrophages and T cells. These findings underscore the potential of targeting immune evasion mechanisms in high mRNAsi tumor cells as part of a precision medicine approach.

In this study, we identified BOP1, DSE, PSMD10, SRPK1, and CTBP1 as key prognostic genes in LMS. Existing literature provides strong evidence for their oncogenic or regulatory roles in various cancers. BOP1 is widely recognized as an oncogene in hepatocellular carcinoma and several other cancers, where it promotes epithelial-to-mesenchymal transition (EMT) and correlates with poor prognosis across pan-cancer cohorts (51–53). DSE has been reported to act as a tumor suppressor in melanoma by regulating extracellular matrix interactions and immune cell infiltration (52, 54). PSMD10 contributes to tumorigenesis through proteasomal regulation and has been implicated in liver and thyroid cancers (52, 55). SRPK1 facilitates tumor progression in gastric and breast cancers by modulating RNA splicing and has also shown elevated expression in certain sarcoma subtypes, including synovial sarcoma (56, 57). CTBP1, a transcriptional co-repressor, is involved in EMT and tumor plasticity and has been linked to sarcomatoid transformation in liver cancer (58, 59). While these genes have been extensively studied in other malignancies, their specific roles in LMS remain largely unexplored. Our findings provide new evidence for their clinical relevance in soft tissue sarcomas and support further investigation into their biological functions and therapeutic potential in LMS.

### 4.2 Functional validation of prognostic genes

To confirm the functional roles of the identified prognostic genes, we conducted a series of experimental validations to assess their impact on tumor cell migration, invasion, and colony formation. Scratch assays demonstrated that silencing BOP1, CTBP1, DSE, PMSD10, and SRPK1 significantly reduced LMS cell migration compared to control cells. Similarly, Transwell migration assays confirmed a significant reduction in cell motility upon knockdown of these genes, reinforcing their role in tumor cell invasiveness. Moreover, colony formation assays showed that knockdown of these genes led to a marked decrease in colony formation ability, suggesting their involvement in tumor cell proliferation and survival.

These functional experiments provide strong evidence that BOP1, CTBP1, DSE, PMSD10, and SRPK1 play key roles in LMS progression, acting as potential therapeutic targets to limit tumor invasion and metastasis. The results also support the hypothesis that high-stemness tumor cells rely on these genes to maintain aggressive phenotypes.

### 4.3 Future directions

Although our study provides important insights into the molecular mechanisms underlying LMS progression, several avenues for future research remain. Functional validation of the six prognostic genes identified in this study is critical to confirm their role in tumor progression and immune modulation. Experimental models, including knockdown or overexpression studies and *in vivo* validation, would help establish the causal relationship between these genes and LMS invasiveness.

Moreover, the observed complexity of the immune microenvironment in high mRNAsi tumors calls for further investigation into the potential for immune checkpoint inhibitors and combination therapies. Specifically, targeting immune evasion mechanisms, such as TGFβ and EGF signaling pathways, could offer novel therapeutic strategies to improve treatment outcomes for LMS patients. In addition, future studies should focus on large-scale clinical validation to assess the generalizability of the stemness risk score and the prognostic model developed in this study. Longitudinal cohort studies would be crucial in evaluating whether the risk genes and immune microenvironment features identified here can be used for early detection, monitoring treatment response, and predicting clinical outcomes in LMS patients. Although current clinical trials have shown limited efficacy of PD-1/PD-L1 inhibitors in LMS, with objective response rates below 10% and low PD-L1 expression across most tumors, our findings suggest that high-stemness LMS cells exhibit a more complex and active tumor immune microenvironment. This implies that certain malignant subpopulations may retain immune-interactive potential (60–63).

Finally, integrating multi-omics data, including genomic, epigenomic, and proteomic data, with single-cell sequencing could provide a more comprehensive understanding of the tumor microenvironment and its role in LMS metastasis. This will help uncover additional therapeutic targets and facilitate the development of more effective, personalized treatments for LMS.

### 4.4 Limitations

This study has several limitations. First, although we integrated multiple publicly available datasets, the number of LMS cases in the single-cell RNA sequencing (scRNA-seq) cohort was limited, with only four samples derived from the GSE212527 dataset. The small sample size may reduce the statistical power and limit the generalizability of our findings across the broader LMS patient population. Second, scRNA-seq data are inherently affected by batch effects, especially when generated using different platforms, sequencing depths, or protocols. While we employed the Harmony algorithm to correct for these variations, residual batch-specific noise may still influence cell clustering and downstream analyses. Third, the prognostic model was constructed using FPKM values from the TCGA-SARC cohort without internal or external validation, which could impact its robustness and predictive accuracy. Future studies with larger cohorts, cross-platform validation, and experimental validation will be essential to further substantiate our findings.

## 5 Conclusion

This study identified key prognostic markers and immune characteristics of leiomyosarcoma (LMS) by integrating single-cell RNA sequencing (scRNA-seq) and bulk RNA-seq data. We found that high-stemness LMS cells exhibit greater invasiveness, a more complex immune microenvironment, and stronger cell-cell interactions, particularly through TGFβ and EGF signaling pathways. Six prognostic genes (BOP1, CTBP1, DSE, PSMD10, SRPK1, and HACD4) were identified, with a stemness risk score model demonstrating strong predictive power for patient prognosis. Functional validation experiments confirmed that BOP1, CTBP1, DSE, PMSD10, and SRPK1 promote LMS cell migration, invasion, and colony formation, further highlighting their role in tumor progression. These findings provide new insights into tumor progression and immune regulation in LMS and highlight potential therapeutic targets for precision medicine. Future research should focus on clinical validation, immune-based therapies, and targeted inhibition of key pathways, which could pave the way for improved treatment strategies for LMS patients.

## Data Availability

The original contributions presented in the study are included in the article/**Supplementary Material**. Further inquiries can be directed to the corresponding authors.
