# Supplementary material for: Integrated single-cell and bulk RNA-seq analysis reveals prognostic stemness genes in leiomyosarcoma
Source: Front Oncol. 2025 Sep 2;15:1604413. doi: 10.3389/fonc.2025.1604413 (PMC12436121; doi:10.3389/fonc.2025.1604413)
Supplement: Supplementary file 1 [file DataSheet1.pdf]

Table S1 siRNA Sequences

Gene	sense（5'-3'）	antisense（5'-3'）
siBOP1-1	GCAAGAUGAGGGUGAAUGUAG	ACAUUCACCCUCAUCUUGCGC
siBOP1-2	GGAUGCUGAGACACCACAAGA	UUGUGGUGUCUCAGCAUCCUG
siBOP1-3	CAGUGAGGAUGAUGACGAAGG	UUCGUCAUCAUCCUCACUGCU
siCTBP1-1	CAGAAGAAGUCAGUAGUUAUU	UAACUACUGACUUCUUCUGCU
siCTBP1-2	CUAGCAAUAUUUUGGUUAAAA	UUAACCAAAAUAUUGCUAGCC
siCTBP1-3	GCUUCAACGUGCUCUUCUACG	UAGAAGAGCACGUUGAAGCCG
siDSE-1	GTGTGTTTTTCATATATTTGC	AAAUAUAUGAAAAACACACUG
siDSE-2	GAAUCAAGGAUAUCUUCAAGA	UUGAAGAUAUCCUUGAUUCAU
siDSE-3	CCUACAAUGGGACAAACUAUG	UAGUUUGUCCCAUUGUAGGGA
siPMSD10-1	GGAAGAGTTGAAGGAGAGT	AGGACTCCTTCATTCCTCTC
siPMSD10-2	GAGGAGTATTCTGGCCGATAAAT	ATTCGGAAGTAAAGACCATTTA
siPMSD10-3	CTGGGTCACCTCGGGACTCGC	GAGTCCAGGTGAGGCCAGCGT
siSRPK1-1	GCAAGAAGAUCCUAAUGAUUA	AUCAUUAGGAUCUUCUUGCUC
siSRPK1-2	CUAAACCAGCUGACAAAAUGU	AUUUUGUCAGCUGGUUUAGGC
siSRPK1-3	GGAAUUUUUCACCAAAAAAGG	UUUUUUGGUGAAAAAUUCCUU

Table S2 Primer Sequences

Gene	Forward Primer（5'-3'）	Reverse Primer（5'-3'）
BOP1	GTGGGCTTCAACCCCTATGAG	CCATGCGAGAGACCTTCTCC
CTBP1	AAAGCCCTCCGCATCATCG	AGACGGCAATGCCTAAATCCC
DSE	CCCTTGGAATACCTCCCTCC	CCATGTCTCGGGCTTCAATGT
PMSD10	GGGTGTGTGTCTAACCTAATGG	GGCCAGAATACTCTCCTTCAACT
SRPK1	ATGGAGCGGAAAGTGCTTG	GAGCCTCGGTGCTGAGTTT
GAPDH	GGAGCGAGATCCCTCCAAAAT	GGCTGTTGTCATACTTCTCATGG

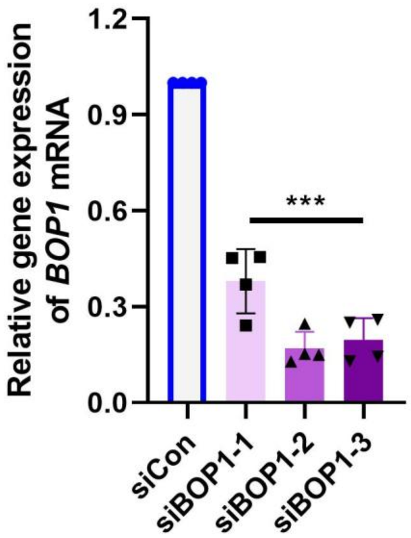

Figure S1. Relative gene expression of BOP1 mRNA following siRNA-mediated knockdown. Data represent the mean ± SD. Statistical significance is indicated by

asterisks: ***p < 0.001.

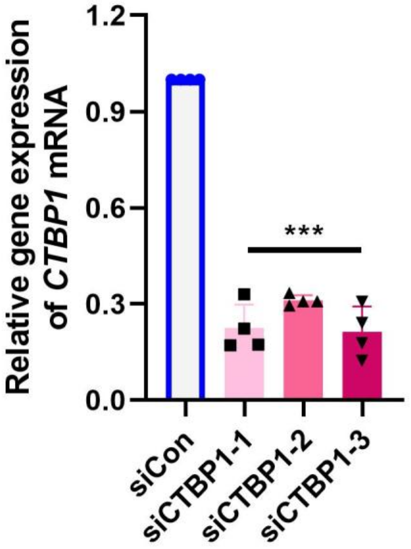

Figure S2. Relative gene expression of CTBP1 mRNA following siRNA-mediated knockdown. Data represent the mean ± SD. Statistical significance is indicated by asterisks: ***p < 0.001.

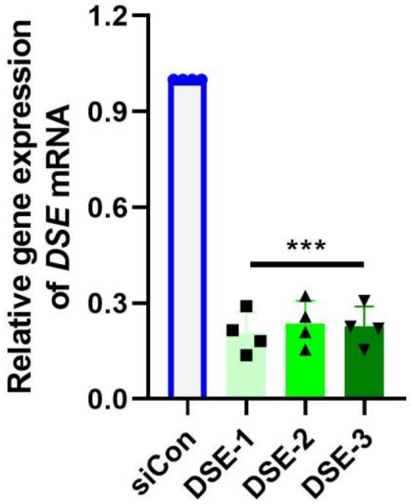

Figure S3. Relative gene expression of DSE mRNA following siRNA-mediated knockdown. Data represent the mean ± SD. Statistical significance is indicated by asterisks: ***p < 0.001.

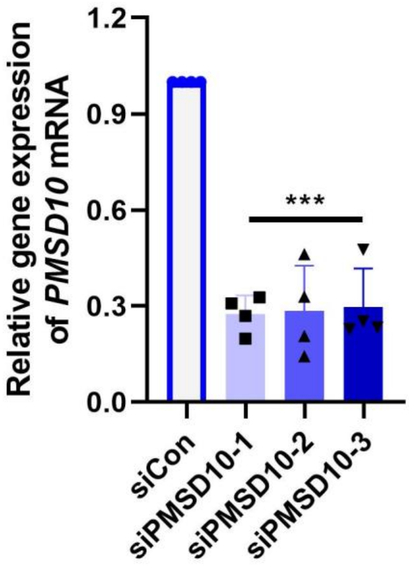

Figure S4. Relative gene expression of PMSD10 mRNA following siRNA-mediated knockdown. Data represent the mean ± SD. Statistical significance is indicated by asterisks: ***p < 0.001.

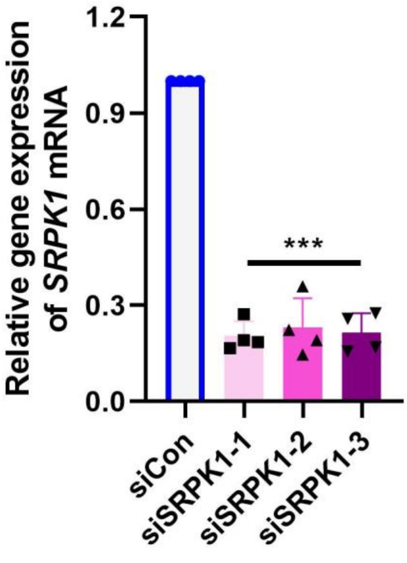

Figure S5. Relative gene expression of SRPK1 mRNA following siRNA-mediated knockdown. Data represent the mean ± SD. Statistical significance is indicated by asterisks: ***p < 0.001.
